# Circuit Depth Reduction for Gate-Model Quantum Computers

**DOI:** 10.1038/s41598-020-67014-5

**Published:** 2020-07-08

**Authors:** Laszlo Gyongyosi, Sandor Imre

**Affiliations:** 1 School of Electronics and Computer Science, University of Southampton, Southampton, SO17 1BJ UK; 2 Department of Networked Systems and Services, Budapest University of Technology and Economics, Budapest, H-1117 Hungary; 3 MTA-BME Information Systems Research Group, Hungarian Academy of Sciences, Budapest, H-1051 Hungary

**Keywords:** Mathematics and computing, Computer science, Pure mathematics

## Abstract

Quantum computers utilize the fundamentals of quantum mechanics to solve computational problems more efficiently than traditional computers. Gate-model quantum computers are fundamental to implement near-term quantum computer architectures and quantum devices. Here, a quantum algorithm is defined for the circuit depth reduction of gate-model quantum computers. The proposed solution evaluates the reduced time complexity equivalent of a reference quantum circuit. We prove the complexity of the quantum algorithm and the achievable reduction in circuit depth. The method provides a tractable solution to reduce the time complexity and physical layer costs of quantum computers.

## Introduction

Gate-model quantum computers are realized by unitary operators (quantum gates) and quantum states^1–29^. As the technological limits of current semiconductor technologies will be reached within the next few years^30–40^, fundamentally different solutions provided by quantum technologies will be significant in the experimental realization of future computations^15–18,31,32,41–72^. A quantum computer is set up with a quantum gate structure, that is, via a set of unitary operators. These quantum gates can realize different quantum operations and can be defined on different numbers of input quantum states^15–18,41–43,45,52,53^. In a quantum computer environment, the depth of the quantum gate structure refers to the number of time steps (time complexity) required for the quantum operations making up the circuit to run on the quantum hardware^15–18,41–43,45,52–59^. A crucial problem here is the time complexity reduction of the quantum gate structure of the quantum computer. Practically, this problem is such that an equivalent quantum state of the output quantum state of the original the reference quantum circuit (e.g., non-reduced time complexity circuit) can be obtained using a reduced time complexity quantum gate structure. Particularly, currently there exists no plausible and implementable solution for the time complexity reduction of quantum computers. Gate-model quantum computer implementations are affected by the problem of high time complexities and a universal (i.e., platform independent) and tractable solution for the time complexity reduction is essential. Relevant implication of this problem is the high economic cost of the physical apparatuses required for experimentally implementing practical quantum computation: specifically, the high economic cost of the high-precision quantum hardware elements required in the implementation of high-performance quantum circuits.

The quantum circuit of the quantum computer is modeled as an arbitrary quantum circuit with an arbitrary circuit depth formulated via a unitary sequence of *L* unitary operators. Each unitary is set via a particular Pauli operator and gate parameter (see also Section 2 for a detailed description). The input problem fed into the quantum computer is an arbitrary computational problem ${\mathscr{P}}$ with an objective function *C*. The *C* objective function is a subject of maximization via the quantum computer, i.e., via the unitaries of the circuit structure of the quantum computer. The objective function can model arbitrary combinatorial optimization problems^9,10,42^, large-scale programming problems^10^ such as the graph coloring problem, molecular conformation problem, job-shop scheduling problem, manufacturing cell formation problem, or the vehicle routing problem^10^. For a detailed description of input problems, we suggest^2–4,8–10,42–45^.

Another important application of gate-model quantum computations is the near-term quantum devices of the quantum Internet^20,30,36–39,46–49,59,61,62,73–93^.

Here, we define a quantum algorithm for the time complexity reduction of any quantum circuit of quantum computers set up with an arbitrary number of unitary gates. The aim of the proposed framework is to reduce the time complexity of an arbitrary reference quantum circuit and a maximization of the objective function of the computational problem fed into the quantum computer. The method defines the reduced time complexity equivalent of the reference quantum circuit and recovers the reference output quantum state via the reduced time complexity quantum circuit (Note: the terminology of quantum state refers to an input or output quantum system, while the terminology of quantum gate refers to a unitary operator.). The reduced structures are determined via a pre-processing phase in the logical layer, and only the reduced time complexity quantum circuit and reduced quantum state are implemented in the physical layer. The pre-rocessing phase integrates a machine learning^94–97^ unit for the parameter optimization. The high complexity reference quantum circuit and reference quantum input are characterized only in the pre-processing phase without any physical level implementation. The framework applies a quantum algorithm on the output of the reduced quantum gate structure to recover the equivalent quantum state of the output quantum state of the non-reduced reference structure. In particular, the proposed framework and the defined quantum algorithm are universal since they have no requirements for the structure of the reference (e.g., non-reduced) quantum circuit subject to be reduced, for the number of unitaries in the reference structure, for the size of the input quantum state of the reference quantum circuit, nor for the dimensions of the actual quantum state. The quantum algorithm is defined as a fixed, auxiliary hardware component for an arbitrary quantum computer environment, with a pre-determined constant computational complexity as an auxiliary cost of the application of the algorithm. Specifically, we prove that the auxiliary cost of the proposed quantum algorithm is orders lower than the reachable amount of the reduction in time complexity, and the computational cost of the quantum algorithm becomes negligible in practice. The method also allows significantly reducing the economic cost of physical layer implementations, since the required elements and high-cost hardware components can be reduced in an experimental setting.

The novel contributions of our manuscript are as follows:

1. We define a quantum algorithm for circuit depth reduction of quantum circuits of gate-model quantum computers.
2. We define the computational cost of the proposed quantum algorithm and prove that it is significantly lower than the gainable reduction in time complexity.
3. The algorithm provides a tractable solution to reduce circuit depth and the economic cost of implementing the physical layer quantum computer by reducing quantum hardware elements.
4. The results are useful for experimental gate-model quantum computations and near-term quantum devices of the quantum Internet.

This paper is organized as follows. Section 2 defines the system model. Section 3 proposes the quantum algorithm and proves the computational complexity. Section 4 discusses the performance of the algorithm. Finally, Section 5 concludes the results. Supplemental material is included in the Appendix.

## System Model

Let *QG*_0_ be a reference quantum circuit (quantum gate structure) with a sequence of *L* unitaries^42^, defined as

1 $$U(\vec{\theta })={U}_{L}({\theta }_{L}){U}_{L-1}({\theta }_{L-1})\ldots {U}_{1}({\theta }_{1}),$$

where $\vec{\theta }$ is the *L*-dimensional vector of the gate parameters of the unitaries (gate parameter vector),

2 $$\vec{\theta }={({\theta }_{1},\ldots ,{\theta }_{L})}^{T},$$

and an *i*-th unitary gate *U*_*i*_(*θ*_*i*_) is evaluated as

3 $${U}_{i}({\theta }_{i})=\exp \,(\,-\,i{\theta }_{i}{P}_{i}),$$

where *P*_*i*_ is a generalized Pauli operator acting on a few quantum states (qubits in an experimental setting) formulated by the tensor product of Pauli operators {*σ*_*x*_, *σ*_*y*_, *σ*_*z*_}^42^. Note, that $U(\vec{\theta })$ in (1) identifies a unitary resulted from the serial application of the *L* unitary operators *U*_*L*_(*θ*_*L*_)*U*_*L*−1_(*θ*_*L*−1_) … *U*_1_(*θ*_1_), and for an input quantum state |*φ*〉,

4 $$U(\vec{\theta })|\varphi \rangle ={U}_{L}({\theta }_{L}){U}_{L-1}({\theta }_{L-1})\ldots {U}_{1}({\theta }_{1})|\varphi \rangle .$$

A qubit system example with a sequence of *L* unitaries is as follows. Let assume that the *QG*_0_ structure of the quantum computer consists of *g* single-qubit and *m* two-qubit unitaries, *L* = *g* + *m*, such that a *j*-th single-qubit gate realizes an ${X}_{j}={\sigma }_{x}^{j}$ operator, while a two-qubit gate realizes a ${Z}_{j}{Z}_{k}={\sigma }_{z}^{j}{\sigma }_{z}^{k}$ operator (see also^42^). Then, at a particular objective function *C* of an arbitrary computational problem subject of a maximization via the quantum computer, the $U(\vec{\theta })$ sequence from (1) can be rewritten as

5 $$U(\vec{\theta })=U(B,\vec{\beta })U(C,\vec{\gamma }),$$

where

6 $$U(B,\vec{\beta })=\prod _{j}\,U({B}_{j},{\beta }_{j}),$$

where $\vec{\beta }$ is the gate parameter vector of the *g* single-qubit unitaries,

7 $$\vec{\beta }={({\beta }_{1},\ldots ,{\beta }_{g})}^{T},$$

while *B* is defined as

8 $$B=\sum _{j}\,{X}_{j}$$

with

9 $${X}_{j}={\sigma }_{x}^{j},$$

and

10 $$U({B}_{j},{\beta }_{j})=\exp (-i{\beta }_{j}{X}_{j}),$$

where *B*_*j*_ = *X*_*j*_, while the two-qubit unitaries are defined as

11 $$U(C,\vec{\gamma })=\prod _{\langle jk\rangle \in Q{G}_{0}}\,U({C}_{jk},{\gamma }_{jk}),$$

where 〈*jk*〉 ∈ *QG*_0_ is a physical connection between qubits *j* and *k* in the hardware-level of the *QG*_0_ structure of the quantum computer, $\vec{\gamma }$ is the gate parameter vector of the *m* two-qubit unitaries

12 $$\vec{\gamma }={({\gamma }_{1},\ldots ,{\gamma }_{m})}^{T},$$

while

13 $$C=\sum _{\langle jk\rangle \in Q{G}_{0}}\,{C}_{jk},$$

where *C*_*jk*_ is a component of the objective function, while unitary *U*(*C*_*jk*_, *γ*_*jk*_) for a given 〈*jk*〉 is defined as

14 $$U({C}_{jk},{\gamma }_{jk})=U({Z}_{j}{Z}_{k},{\gamma }_{jk}{C}_{jk})=\exp (-i{\gamma }_{jk}{C}_{jk}{Z}_{j}{Z}_{k}),$$

where

15 $${Z}_{j}{Z}_{k}={\sigma }_{z}^{j}{\sigma }_{z}^{k}.$$

At a particular physical connectivity of *QG*_0_, the objective function *C* therefore can be written as

16 $$C(z)=\sum _{\langle jk\rangle \in Q{G}_{0}}\,{C}_{jk}(z),$$

where *C*_*jk*_(*z*) is the objective function component evaluated for a given 〈*jk*〉, as

17 $${C}_{\langle jk\rangle }(z)=\frac{1}{2}(1-{z}_{i}{z}_{j}),$$

while *z* is an *N*-length input bitstring,

18 $$z={z}_{1}{z}_{2}\ldots {z}_{N},$$

where *z*_*i*_ identifies an *i*-th bit, *z*_*i*_ ∈ {−1, 1}.

For a given *z*, a |*z*〉 computational basis state is defined as

19 $$|z\rangle =|{z}_{1}{z}_{2}\ldots {z}_{N}\rangle ,$$

and the |*ϕ*〉 output system of *QG*_0_ is as

20 $$|\phi \rangle =U(\vec{\theta })|z\rangle ,$$

that can be evaluated further via (6) and (11), as

21 $$\begin{array}{rcl}|\phi \rangle  & = & U(B,\vec{\beta })U(C,\vec{\gamma })|z\rangle \\  & = & \prod _{j}\,U({B}_{j},{\beta }_{j})\prod _{\langle jk\rangle \in Q{G}_{0}}\,U({C}_{jk}(z),{\gamma }_{jk})|z\rangle \\  & = & \prod _{j}\,\exp (-i{\beta }_{j}{X}_{j})\prod _{\langle jk\rangle \in Q{G}_{0}}\,\exp (-i{\gamma }_{jk}{C}_{jk}(z){Z}_{j}{Z}_{k})|z\rangle .\end{array}$$

To achieve the quantum parallelism, the input system |*φ*〉 = |*X*〉 of the quantum computer is set as an *N*-length *d*-dimensional (*d* = 2 for a qubit system) quantum state in the superposition of all possible *d*^*N*^ states. For a qubit system, it means that input |*X*〉 is an *N*-qubit quantum state in a superposition of all possible 2^*N*^ states |0〉 to |2^*N*^ − 1〉, and the computations are performed on 2^*N*^ states in parallel in the quantum computer.

Let |*X*〉 be a superposed input system of the non-reduced *QG*_0_ gate structure:

22 $$|X\rangle =\frac{1}{\sqrt{{d}^{N}}}\mathop{\sum }\limits_{{x}_{i}=1}^{{d}^{N}}\,|{x}_{i}\rangle ,$$

where |*x*_*i*_〉 is an *i*-th input state (represented as an *N*-length bit string for a qubit system), *i* = 1, …, *n*, *n* = *d*^*N*^, of the *QG*_0_ structure of the quantum computer.

To describe the parallel processing of the *n* input vectors of |*X*〉 (see (22)), {|*x*_1_〉, …, |*x*_*n*_〉} of |*X*〉 (see (22)) in the quantum computer, let ${\vec{\theta }}_{i}$ be the gate parameter vector associated with a given |*x*_*i*_〉 of |*X*〉:

23 $${\vec{\theta }}_{i}={({\theta }_{i,1},\ldots ,{\theta }_{i,L})}^{T}.$$

Let *X* be the classical representation of |*X*〉 in (22) to get

24 $$X=\frac{1}{\sqrt{{d}^{N}}}{({x}_{1},\ldots ,{x}_{n})}^{T},$$

where *x*_*i*_ is the classical representation of |*x*_*i*_〉. (Note, that *X* and *x*_*i*_ are not accessible in the quantum computer, since the quantum algorithm operates in the quantum regime on quantum states. The classical representation is used only as an abstracted auxiliary representation to describe the steps of the algorithm in a plausible manner).

Then, let ${U}_{0}(\vec{\theta })$ be the non-reduced gate structure matrix of *QG*_0_:

25 $${U}_{0}(\vec{\theta })=({U}_{0}({\vec{\theta }}_{n}){U}_{0}({\vec{\theta }}_{n-1})\ldots {U}_{0}({\vec{\theta }}_{1})),$$

where

26 $$\vec{\theta }=({\vec{\theta }}_{1},\ldots ,{\vec{\theta }}_{n})$$

and ${U}_{0}({\vec{\theta }}_{i})$ is the unitary sequence associated with |*x*_*i*_〉 in *QG*_0_, defined as

27 $${U}_{0}({\vec{\theta }}_{i})={U}_{L}({\theta }_{i,L}){U}_{L-1}({\theta }_{i,L-1})\ldots {U}_{1}({\theta }_{i,1}).$$

At an *n*-dimensional output vector

28 $$Y=\frac{1}{\sqrt{{d}^{N}}}{({y}_{1},\ldots ,{y}_{n})}^{T},$$

and the |*Y*〉 output quantum state of the non-reduced *QG*_0_ structure is

29 $$|Y\rangle ={U}_{0}(\vec{\theta })|X\rangle =\frac{1}{\sqrt{{d}^{N}}}\sum _{{y}_{i}}\,|{y}_{i}\rangle =\frac{1}{\sqrt{{d}^{N}}}\mathop{\sum }\limits_{{x}_{i}=1}^{{d}^{N}}\,{U}_{0}({\vec{\theta }}_{i})|{x}_{i}\rangle .$$

To define the reduced gate structure, QG^*^, it is necessary to find a reduced $U({\vec{\theta }}_{i}^{{\prime} })$ with a reduced input $|{\tilde{x}}_{i}\rangle$, for all *i*.

Then, let $\tilde{X}$ be the classical representation of the reduced quantum state $|\tilde{X}\rangle$ fed into QG^*^, as

30 $$\tilde{X}=\frac{1}{\sqrt{{n}^{\ast }}}{({\tilde{x}}_{1},\ldots ,{\tilde{x}}_{{n}^{\ast }})}^{T}$$

and

31 $$|\tilde{X}\rangle =\frac{1}{\sqrt{{n}^{\ast }}}\mathop{\sum }\limits_{{\tilde{x}}_{i}=1}^{{d}^{{N}^{\ast }}}|{\tilde{x}}_{i}\rangle ,$$

where N^*^ is the number of *d*-dimensional (physical) quantum states that formulate $|\tilde{X}\rangle$, n^*^ = *d*^N*^, while the unitaries $U(\vec{\theta }{\prime} )$ of QG^*^ are

32 $$U(\vec{\theta }{\prime} )=(U({\vec{\theta }}_{{n}^{\ast }}^{{\prime} })U({\vec{\theta }}_{{n}^{\ast }-1}^{{\prime} })\ldots U({\vec{\theta }}_{1}^{{\prime} })).$$

where

33 $$\vec{\theta }{\prime} =({\vec{\theta }}_{1}^{{\prime} },\ldots ,{\vec{\theta }}_{{n}^{\ast }}^{{\prime} })$$

and $U({\vec{\theta }}_{i}^{{\prime} })$ is the reduced unitary sequence associated with $|{\tilde{x}}_{i}\rangle$, defined as

34 $$U({\vec{\theta }}_{i}^{{\prime} })={U}_{L}({\tilde{\theta }}_{i,L}){U}_{L-1}({\tilde{\theta }}_{i,L-1})\ldots {U}_{1}({\tilde{\theta }}_{i,1}).$$

The pre-processing phase determines output *Z* of QG^*^ as a classical representation

35 $$Z=U(\vec{\theta }{\prime} )\tilde{X},$$

and the output quantum state |*Z*〉 of QG^*^ therefore yields

36 $$|Z\rangle =U(\vec{\theta }{\prime} )|\tilde{X}\rangle .$$

The notations of the system model are also summarized in Table A.1 of the Supplemental Information.

### Problem statement

Problems 1–3 summarize the problems to be solved.

#### **Problem 1**

*Find a classical pre-processing* ${\mathscr{P}}$ *for calculating the* $|\tilde{X}\rangle$ reduced input system and the gate parameters of the QG* *reduced time complexity gate structure*.

#### **Problem 2**

*Find a universal (independent of the number L of the unitaries in QG*_0_*) unitary operator U*_*R*_ *with a set* ${\mathscr{R}}$ *of quantum registers to recover output* |*Y*〉 *of the non-reduced QG*_0_ *structure from output* |*Z*〉 of the QG* *reduced time complexity gate structure*.

#### **Problem 3**

*Determine the time complexity of U*_*R*_ and the reduction in the overall time complexity of QG*.

Theorems 1–3 give the resolutions of Problems 1–3.

**Figure 1:**
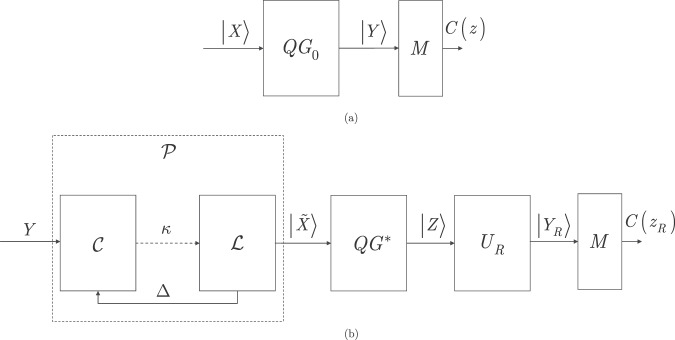
(**a**) The non-reduced time complexity quantum circuit *QG*_0_ (reference circuit) with an input quantum state |*X*〉. The output of *QG*_0_ is |*Y*〉. The state |*Y*〉 is measured via a measurement *M* to get the classical string *z* to evaluate the objective function *C*(*z*). (**b**) System model of the time complexity reduction scheme. Pre-processing phase ${\mathscr{P}}$: the *Y* classical representation of output |*Y*〉 of *QG*_0_ is pre-processed by the pre-processing unit ${\mathscr{P}}$. Unit ${\mathscr{P}}$ contains a ${\mathscr{C}}$ computational block that outputs a vector *κ*, fed into an ${\mathscr{L}}$ machine learning control unit for the Δ error feedback. Unit ${\mathscr{P}}$ outputs $\tilde{X}$ and the gate parameters of the reduced structure that defines QG^*^. Quantum phase: from $\tilde{X}$ and the gate parameters, $|\tilde{X}\rangle$ and QG^*^ are set up. System $|\tilde{X}\rangle$ is fed into the reduced quantum circuit QG^*^. The output of QG^*^ is |*Z*〉, which is fed into the *U*_*R*_ recovery quantum algorithm. The *U*_*R*_ quantum operation outputs the |*Y*_*R*_〉 system, which is the reference output |*Y*〉 of the reference circuit *QG*_0_. The state |*Y*_*R*_〉 is measured via a measurement *M* to get the classical string *z*_*R*_ to evaluate objective function *C*(*z*_*R*_).

The non-reduced time complexity quantum circuit *QG*_0_ (reference circuit) with an input quantum state |*X*〉 is showed in Fig. 1(a). Figure 1(b) depicts the system model for the problem resolution. The method is realized via unitary *U*_*R*_ and ${\mathscr{P}}$ pre-processing, such that *U*_*R*_ is implemented in the physical layer, while ${\mathscr{P}}$ is only a logical-layer process. Only the reduced input quantum state $|\tilde{X}\rangle$ and the reduced quantum gate structure QG^*^ must be built up in the physical layer to yield the reference output system |*Y*〉 of the reference circuit *QG*_0_ via |*Y*_*R*_〉. In both cases, the output states are measured via a measurement *M* to get a classical bitring for the objective function evaluation. As a next step, the gate parameter values of the unitaries of the circuits are calibrated until an optimal objective function value is not reached. The calibration of the gate parameters is a separate optimization procedure and its aim is fundamentally differ from the aim of ${\mathscr{P}}$, and therefore it is not part of the circuit depth reduction method. Note that existing algorithms can be utilized for this task (such as a the algorithms proposed in^19^ and^20^, or some gradient independent methods^98^).

### Pre-processing

#### **Theorem 1**

*There exists a* ${\mathscr{P}}$ *pre-processing to determine the* $|\tilde{X}\rangle$ *input system and the* $U({\vec{\theta }}_{i}^{{\prime} })$ *gate parameters*, *i* = , …, *n*, for the reduced QG* *gate structure for an arbitrary non-reduced QG*_0_ *structure with* $U({\vec{\theta }}_{i})$ *and input* |*X*〉.

**Proof**. The ${\mathscr{P}}$ pre-processing phase can be decomposed as ${\mathscr{P}}={\mathscr{C}}{\mathscr{L}}$, where ${\mathscr{C}}$ is a computational block, while ${\mathscr{L}}$ is a machine learning control block to calibrate the results of ${\mathscr{C}}$. We first define block ${\mathscr{C}}$, then discuss ${\mathscr{L}}$. The ${\mathscr{P}}$ pre-processing is a procedure to stabilize the output of the reduced quantum circuit. ${\mathscr{P}}$ is defined between the components ${\mathscr{C}}$ and ${\mathscr{L}}$ to evaluate $|\tilde{X}\rangle$ and to set the gate parameters of the reduced quantum circuit structure QG^*^ using the reference output |*Y*〉 of *QG*_0_. Note, as the output |*Y*_*R*_〉 is fed into an *M* measurement block, the measurement results provide a feedback to calibrate ${\mathscr{P}}$ in every subroutine of the protocol to produce a final saturated output. The Δ output of the ${\mathscr{L}}$ machine learning control unit is used as a feedback in unit ${\mathscr{C}}$. For the definition of Δ, see (116) in Algorithm 1.

In the ${\mathscr{C}}$ computational block, the reduced $U({\vec{\theta }}_{i}^{{\prime} })$ and ${\tilde{x}}_{i}$ are determined for ∀*i*, in the following manner. Note, since ${\mathscr{P}}$ outputs the parameters of the reduced quantum gate structure, the extra complexity of a quantum structure can be replaced with classical complexity in the form of machine learning in the proposed framework.

Operation ${\mathscr{C}}$ sets a one-dimensional discrete cosine transform^99^ in the reduction method, thus a matrix *G* is defined as a generator matrix to evaluate the output coefficients of ${\mathscr{C}}$, see later (45). The definition of ${\mathscr{C}}$ (see later in (40)) comes from the fact that any *U* unitary operator can be rewritten via the cos and sin functions, and using cosine functions rather than sine functions is critical for a compression^99^. In our setting, this is because fewer cosine functions are needed to approximate a particular *U* unitary operator.

Let *x*_*i*_ be the classical representation of |*x*_*i*_〉, and ${y}_{i}=U({\vec{\theta }}_{i}){x}_{i}$ be the classical representation of |*y*_*i*_〉. Using the sequences of the *L* unitaries in (29), define a matrix *G* with *n* coefficients *a*_*i*_, *i* = 1, …, *n*, as

37 $$\begin{array}{rcl}G & = & {(\begin{array}{ccc}{a}_{1} & \cdots  & {a}_{n}\end{array})}^{T}\\  & = & (\begin{array}{c}(\Sigma \,{\theta }_{1})+{x}_{1}\\ (\Sigma \,{\theta }_{2})+{x}_{2}\\ \vdots \\ (\Sigma \,{\theta }_{n})+{x}_{n}\end{array})=(\begin{array}{c}({\Sigma }_{j=1}^{L}\,{\theta }_{1,j})+{x}_{1}\\ ({\Sigma }_{j=1}^{L}\,{\theta }_{2,j})+{x}_{2}\\ \vdots \\ ({\Sigma }_{j=1}^{L}\,{\theta }_{n,j})+{x}_{n}\end{array}),\end{array}$$

where

38 $$\Sigma \,{\theta }_{i}=\underset{j=1}{\overset{L}{\Sigma }}\,{\theta }_{i,j},$$

where *θ*_*i*,*j*_ identifies the gate parameter of a *j*-th unitary *U*_*i*,*j*_(*θ*) associated to an *i*-th input *x*_*i*_, while unitary sequence ${U}_{0}({\vec{\theta }}_{i})$ to an *i*-th input *x*_*i*_ is

39 $${U}_{0}({\vec{\theta }}_{i})=\exp (i\varSigma \,{\theta }_{i}{P}_{i}),$$

where *P*_*i*_ is a generalized Pauli operator.

First, the ${\mathscr{C}}$ operation (one-dimensional discrete cosine transform^99^) is applied to the input matrix *G* from (37),

40 $${\mathscr{C}}\,:\,\{{c}_{p},{f}_{i}\},0\le p,i\le n-1,$$

where *c*_*p*_ is the *p*-th coefficient of ${\mathscr{C}}$,

41 $${c}_{p}=\mathop{\sum }\limits_{i=0}^{n-1}\,{A}_{p}{f}_{i}\,\cos \,\frac{\pi (2i+1)p}{2n},$$

where

42 $${f}_{i}=\mathop{\sum }\limits_{i=0}^{n-1}\,{A}_{p}{c}_{p}\,\cos \,\frac{\pi (2i+1)p}{2n},$$

and *A*_*p*_ is

43 $${A}_{p}=\{\begin{array}{l}\frac{1}{\sqrt{n}},\,{\rm{if}}\,p=0\\ \sqrt{\frac{2}{n}},\,{\rm{if}}\,1 < p < n\end{array}.$$

The coefficients of ${\mathscr{C}}$ defines matrix *γ* as

44 $$\begin{array}{rcl}\gamma  & = & \chi G\\  & = & {({\varsigma }_{1}\cdot G,{\varsigma }_{2}\cdot G,\ldots ,{\varsigma }_{n}\cdot G)}^{T}\\  & = & {({c}_{1},{c}_{2},\ldots ,{c}_{n})}^{T},\end{array}$$

where · is the inner product,

45 $${c}_{i}={\varsigma }_{i}\cdot G=\mathop{\sum }\limits_{k=1}^{n}\,{\varsigma }_{i,k}\times {a}_{k},$$

where coefficients *a*_*k*_-s are given in (37), and *χ* is

46 $$\chi =(\begin{array}{c}{({\varsigma }_{1})}^{T}\\ \vdots \\ {({\varsigma }_{n-1})}^{T}\\ {({\varsigma }_{n})}^{T}\end{array}),$$

where *ς*_*i*_ is an *n*-length vector

47 $${\varsigma }_{i}=({\varsigma }_{i,1},\ldots ,{\varsigma }_{i,n})={A}_{i}{\left(\cos \frac{i\pi }{2n},\cos \frac{3i\pi }{2n},\ldots ,\cos \frac{(2n-1)i\pi }{2n}\right)}^{T}.$$

Then, the *n*-length output vector *κ* of ${\mathscr{C}}$ is defined as

48 $${\mathscr{C}}(Y)=\kappa =\frac{1}{\sqrt{n}}{({\Omega }_{1},\ldots ,{\Omega }_{n})}^{T},$$

where *Y* is given in (28), while Ω_*i*_ is as

49 $$\begin{array}{rcl}{\Omega }_{i} & = & U({\vec{\theta }}_{i}^{{\prime} }){\tilde{x}}_{i}\\  & = & {U}_{L}({\tilde{\theta }}_{i,L}){U}_{L-1}({\tilde{\theta }}_{i,L-1})\ldots {U}_{1}({\tilde{\theta }}_{i,1}){\tilde{x}}_{i}.\end{array}$$

Then, using the coefficients (41), (42) and (43) of ${\mathscr{C}}$, $|{\tilde{x}}_{p}\rangle$ of the reduced state $|\tilde{X}\rangle$ from (31) can be evaluated via the ${\tilde{x}}_{p}$ components of $\tilde{X}$ of (30). A *p*-th input $|{\tilde{x}}_{p}\rangle$ for QG^*^ is defined via (49) as

50 $$|{\tilde{x}}_{p}\rangle =|{\Omega }_{p}{U}^{\dagger }({\vec{\theta }}_{p}^{{\prime} })\rangle =|\mathop{\sum }\limits_{i=0}^{n-1}\,{A}_{p}{x}_{i}\,\cos \,\frac{\pi (2i+1)p}{2n}\rangle ,$$

and the reduced quantum gate sequence $U({\vec{\theta }}_{p}^{{\prime} })$ of $|{\tilde{x}}_{p}\rangle$ in QG^*^, as

51 $$U({\vec{\theta }}_{p}^{{\rm{{\prime} }}})=\exp (i\Sigma {\tilde{\theta }}_{p}{P}_{p}),$$

where *P*_*p*_ is a generalized Pauli operator, and $\Sigma {\tilde{\theta }}_{p}$ is as

52 $$\Sigma {\tilde{\theta }}_{p}=\mathop{\sum }\limits_{i=0}^{n-1}\,{A}_{p}(\Sigma {\theta }_{i})\cos \,\frac{\pi (2i+1)p}{2n}.$$

Therefore, the quantum state |*Z*〉 of QG^*^ is

53 $$|Z\rangle =U(\vec{\theta }{\prime} )|\tilde{X}\rangle =\frac{1}{\sqrt{{d}^{{N}^{\ast }}}}\mathop{\sum }\limits_{{\tilde{x}}_{i}=1}^{{d}^{{N}^{\ast }}}\,U({\vec{\theta }}_{i}^{{\prime} })|{\tilde{x}}_{i}\rangle .$$

The description of the ${\mathscr{L}}$ machine learning control unit is as follows. Unit ${\mathscr{L}}$ uses the results of ${\mathscr{C}}$ to provide feedback for the pre-processing via supervised machine learning control.

The ${\mathscr{L}}$ machine learning algorithm for the pre-processing control is defined in Algorithm 1.

The steps of the ${\mathscr{P}}$ pre-processing method is given in Procedure 1.

■

## Quantum Algorithm

### **Theorem 2**

*The* |*Y*〉 *output of the non-reduced QG*_0_ *structure can be recovered from the output* |*Z*〉 of the reduced structure QG* *via a unitary operator U*_*R*_.

**Proof**. Let $|\tilde{X}\rangle$ be the input quantum state fed into the reduced structure QG^*^, and let |*Z*〉 (see (53)) be the output of the reduced gate structure. The task here is therefore to recover $|Y\rangle ={U}_{0}(\vec{\theta })|X\rangle$ from |*Z*〉. The problem is solved via a unitary *U*_*R*_, as follows.

Without loss of generality, in an *i*-th step, *i* = 1, …, *n*, the goal of the *U*_*R*_ operation is to calculate the quantum state as

54 $$|{\Phi }_{i}\rangle =|{\omega }_{i}\cdot \kappa \rangle ,$$

where *κ* is as in (48), while *ω*_*i*_ = (*ω*_*i*,1_, …, *ω*_*i*,*n*_)^*T*^ is an *n*-length vector defined for a given *j*, as

55 $${\omega }_{j}={\left({e}^{i(\Sigma {\theta }_{1}-\Sigma {\tilde{\theta }}_{1})}{A}_{j}\cos \frac{j\pi }{2n},\ldots ,{e}^{i(\Sigma {\theta }_{n}-\Sigma {\tilde{\theta }}_{n})}{A}_{j}\cos \frac{(2n-1)j\pi }{2n}\right)}^{T},$$

where ∑*θ*_*i*_ is as given in

56 $$\Sigma {\theta }_{i}=\mathop{\sum }\limits_{i=0}^{n-1}\,{A}_{p}(\Sigma {\tilde{\theta }}_{p})\cos \,\frac{\pi (2i+1)p}{2n},$$

where $\Sigma {\tilde{\theta }}_{p}$ is given in (52).

Then let

57 $$W=(\begin{array}{c}{({\omega }_{1})}^{T}\\ \vdots \\ {({\omega }_{n-1})}^{T}\\ {({\omega }_{n})}^{T}\end{array}),$$

such that

58 $$W\kappa ={({\omega }_{1}\cdot \kappa ,\ldots ,{\omega }_{n}\cdot \kappa )}^{T}.$$

Applying *U*_*R*_ for all *i*, yields the recovered quantum state |*Y*_*R*_〉 as

59 $$\begin{array}{rcl}|{Y}_{R}\rangle  & = & \mathop{\sum }\limits_{i=1}^{n}\,|{\Phi }_{i}\rangle =\mathop{\sum }\limits_{i=1}^{n}\,|{\omega }_{i}\cdot \kappa \rangle \\  & = & \frac{1}{\sqrt{n}}\mathop{\sum }\limits_{{x}_{i}=1}^{n}\,{e}^{i(\Sigma {\theta }_{i}-\Sigma {\tilde{\theta }}_{i})}U({\vec{\theta }}_{i}{\prime} )|{x}_{i}\rangle \\  & = & \frac{1}{\sqrt{{d}^{N}}}\mathop{\sum }\limits_{{x}_{i}=1}^{{d}^{N}}\,{U}_{0}({\vec{\theta }}_{i})|{x}_{i}\rangle \\  & = & {U}_{0}(\vec{\theta })|X\rangle ,\end{array}$$

where an *i*-th |*x*_*i*_〉 is as

60 $$|{x}_{i}\rangle =|\mathop{\sum }\limits_{i=0}^{n-1}\,{A}_{p}{\tilde{x}}_{p}\,\cos \,\frac{\pi (2i+1)p}{2n}\rangle ,$$

where *i* ≤ *n* − 1, and *p* ≥ 0, and ${\tilde{x}}_{p}$ is as given in (50); while the ${U}_{0}({\vec{\theta }}_{i})$ gate parameters (see (39)) of the *L* unitaries for a given *i* are evaluated as

61 $$\begin{array}{rcl}{U}_{0}({\vec{\theta }}_{i}) & = & {e}^{i(\Sigma {\theta }_{i}-\Sigma {\tilde{\theta }}_{i})}U({\vec{\theta }}_{i}^{{\prime} })\\  & = & {e}^{i(\Sigma {\theta }_{i}-\Sigma {\tilde{\theta }}_{i})}{e}^{i\Sigma {\tilde{\theta }}_{i}{P}_{i}}\\  & = & {e}^{i\Sigma {\theta }_{i}{P}_{i}}.\end{array}$$

The unitary *U*_*R*_ is defined via a set ${\mathscr{R}}$ of quantum registers as

62 $${U}_{R}\,:\,{\mathscr{R}}=\{|{R}_{1}\rangle ,\ldots ,|{R}_{7}\rangle \},$$

where |*R*_*i*_〉 is the *i*-th quantum register. The registers are initialized via set ${{\mathscr{R}}}_{0}$ as

63 $${{\mathscr{R}}}_{0}=\{\begin{array}{l}|{R}_{1}\rangle =|\partial \rangle ,|{R}_{2}\rangle =|\eta \rangle ,|{R}_{3}\rangle =|i\rangle ,|{R}_{4}\rangle =|\kappa \rangle ,\\ |{R}_{5}\rangle =|0\rangle ,|{R}_{6}\rangle =|0\rangle ,|{R}_{7}\rangle =|0\rangle \end{array}\},$$

where *κ* is given in (48), while ∂ and *η* are initial parameters defined as

64 $$\partial =\frac{{\Vert \kappa \Vert }^{2}}{n},$$

and

65 $$\eta ={\Vert \kappa \Vert }^{2},$$

where

66 $${\Vert \kappa \Vert }^{2}=\kappa \cdot \kappa =\mathop{\sum }\limits_{i=0}^{n-1}\,\frac{1}{n}({\Omega }_{i}\times {\bar{\Omega }}_{i})$$

where $${\Omega }_{i}=U({\vec{\theta }}_{i}^{{\prime} }){\tilde{x}}_{i}$$, and

67 $$\partial \le \frac{1}{n}({y}_{i}\times {y}_{i})\le \eta ,$$

where $${y}_{i}=U({\vec{\theta }}_{i}){x}_{i}$$.

Then, unitary *U*_*R*_ is defined as

68 $${U}_{R}=(2|\psi \rangle \langle \psi |-I){U}_{S},$$

where

69 $$|\psi \rangle =\frac{1}{\sqrt{n}}\mathop{\sum }\limits_{i=0}^{n-1}\,|i\rangle ,$$

and *U*_*S*_ is a unitary defined as

70 $${U}_{S}={({U}_{0})}^{-1}{({O}_{{\Phi }_{i}})}^{-1}{O}_{{f}_{i}}{O}_{{\Phi }_{i}}{U}_{0},$$

with eigenstate

71 $$|{\Psi }_{S}\rangle =|\partial \rangle |\eta \rangle |i\rangle |\kappa \rangle |0\rangle |0\rangle |0\rangle ;$$

where *U*_0_ is an initial unitary operator that prepares state |*R*_5_〉 = |*ω*_*i*_〉 for a given index state |*R*_4_〉 = |*i*〉, where *ω*_*i*_ is given in (55); from an initial |*R*_4_〉|*R*_5_〉 = |*i*〉|0〉 as

72 $${U}_{0}(|i\rangle |0\rangle )=|0\oplus {\omega }_{i}\rangle $$

in the register set ${{\mathscr{R}}}_{0}$ (see (63)), where $\oplus$ is the CNOT operation, while ${O}_{{\Phi }_{i}}$ is an oracle applied on ${{\mathscr{R}}}_{0}$ to compute Φ_*i*_ (54), defined as

73 $$\begin{array}{c}{O}_{{\Phi }_{i}}({{\mathscr{R}}}_{0})={\mathscr{R}}{\prime} \\ :\,\{\begin{array}{l}|{R}_{1}\rangle =|\partial \rangle ,|{R}_{2}\rangle =|\eta \rangle ,|{R}_{3}\rangle =|i\rangle ,|{R}_{4}\rangle =|\kappa \rangle ,\\ |{R}_{5}\rangle =|{\omega }_{i}\rangle ,|{R}_{6}\rangle =|0\rangle ,|{R}_{7}\rangle =|{({\Phi }_{i})}^{2}\rangle \end{array}\},\end{array}$$

where ${\mathscr{R}}{\prime}$ is the resulting register set, while ${O}_{{f}_{i}}$ is an oracle that outputs function *f*_*i*_, as

74 $${O}_{{f}_{i}}\,:\,{f}_{i}=\{\begin{array}{l}1,\,{\rm{if}}\,\partial \le {\left(\frac{1}{\sqrt{n}}{y}_{i}\right)}^{2}\le \eta \\ 0,\,{\rm{otherwise}}\end{array}.$$

Specifically, note that (70) changes only the phase of the state as ${(-1)}^{{f}_{i}}$, where *f*_*i*_ is given in (74), while

75 $${({U}_{0})}^{-1}{({O}_{{\Phi }_{i}})}^{-1}={({O}_{{\Phi }_{i}}{U}_{0})}^{-1}.$$

Applying (74) on (63) yields a register state ${O}_{{f}_{i}}({{\mathscr{R}}}_{0})$ as

76 $${O}_{{f}_{i}}({{\mathscr{R}}}_{0})={(-1)}^{{f}_{i}}{{\mathscr{R}}}_{0},$$

where ${(-1)}^{{f}_{i}}$ is the eigenvalue of *U*_*S*_ in (70).

Then, using the register set (63), let |*ϕ*_0_〉 be the input state for *U*_*R*_ as

77 $$|{\phi }_{0}\rangle =\frac{1}{\sqrt{n}}\mathop{\sum }\limits_{i=0}^{n-1}\,|\partial \rangle |\eta \rangle |i\rangle |\kappa \rangle |0\rangle |0\rangle |0\rangle .$$

Applying (68) *k*-times on (77) yields

78 $$|{\phi }_{k}\rangle ={({U}_{R})}^{k}|{\phi }_{0}\rangle .$$

The *k* iteration number in (78) is a random number, *k* < *c*, where $c=\,{\rm{\min }}\{1.2\cdot m,\sqrt{n}\}$, and *m* is initialized as *m* = 1^99^.

Then let *O*_*Z*_ be an oracle defined on ${\mathscr{R}}{\prime}$ as

79 $$\begin{array}{l}{O}_{Z}({\mathscr{R}}{\prime} )={{\mathscr{R}}}^{\ast }\\ :\,\{\begin{array}{l}|{R}_{1}\rangle =|\partial \rangle ,|{R}_{2}\rangle =|\eta \rangle ,|{R}_{3}\rangle =|i\rangle ,|{R}_{4}\rangle =|\kappa \rangle ,\\ |{R}_{5}\rangle =|{\omega }_{i}\rangle ,|{R}_{6}\rangle =|{\varPhi }_{i}\rangle ,|{R}_{7}\rangle =|0\rangle \end{array}\}.\end{array}$$

Applying *O*_*Z*_*U*_0_ on (78), outputs system state

80 $$|{\phi }^{\ast }\rangle ={O}_{Z}{U}_{0}(|{\phi }_{k}\rangle ).$$

In particular, in system state (80), the state of register |*R*_6_〉 is

81 $$|{R}_{6}\rangle =\mathop{\sum }\limits_{i=1}^{n}\,|{\Phi }_{i}\rangle =|{Y}_{R}\rangle ,$$

therefore yields (59), such that

82 $$\partial \le {({\Phi }_{i})}^{2}\le \eta $$

holds for all *i* of |*Y*_*R*_〉, due to the conditions set in the pre-processing procedure ${\mathscr{P}}$ (see (67)).

Assuming that the input system (77) for *U*_*R*_ is prepared for *R*-times and the output register (81) is measured for *R*-times, i.e., *U*_*R*_ is applied for *R* times in overall, in an *r*-th repetition, *r* = 1, …, *R*, the parameters of the procedure can be valuated as

83 $${\partial }^{(r)}=\frac{{\eta }^{(r)}}{n-{q}^{(r)}},$$

where

84 $${\eta }^{(r)}={\Vert {\kappa }^{(r)}\Vert }^{2}-({\Phi }_{i}^{(r)}\times {\Phi }_{i}^{(r)}),$$

where ${\Phi }_{i}^{(r)}$ is the measured value of |Φ_*i*_〉 in the *r*-th repetition of *U*_*R*_, while *q*^(*r*)^ is the number of coefficients have been already found^99^.

The actual value of *r* requires no increment if the relation

85 $${\tau }^{(r)} > \frac{1}{{\Vert \kappa \Vert }^{2}}{\eta }^{(r)},$$

holds, where *τ*^(*r*)^ is a threshold value in the *r*-th iteration. Otherwise, the value of *r* can be increased, *r* = *r* + 1, as *r* < *R*.

The steps of the quantum algorithm *U*_*R*_ are given in Algorithm 2.

### Distortion measure

As (81) is prepared in Step 4 of *U*_*R*_, the state |*Y*_*R*_〉 can be measured to get the classical string *z*_*R*_ to evaluate objective function *C*(*z*_*R*_), as follows. Measure register |*R*_6_〉 of ${\mathscr{R}}$ via a measurement operator *M* to evaluate objective function *C*(*z*_*R*_), where *z*_*R*_ is a classical string resulted from the measurement of |*Y*_*R*_〉, while *C* is an objective function of an arbitrary computational problem fed into the quantum computer.

The ${\mathscr{D}}$ distortion coefficient associated with the |*Y*_*R*_〉 recovered quantum state (59) can be evaluated at a particular objective function *C*, associated to the computational problem fed into the quantum computer as

86 $${\mathscr{D}}=|C(z)-C({z}_{R})|,$$

where *z* is a classical string resulting from the *M* measurement of |*Y*〉, while *z*_*R*_ is a classical string resulting from the *M* measurement of |*Y*_*R*_〉.

Precisely, assuming *R* measurement rounds, an average of distortion yields

87 $${{\mathscr{D}}}^{(R)}=\frac{1}{R}\mathop{\sum }\limits_{r=1}^{R}\,|{C}^{(r)}(z)-{C}^{(r)}({z}_{R})|,$$

where *C*^(*r*)^(*z*) and *C*^(*r*)^(*z*_*R*_) are the objective function values respectively associated with *z* and *z*_*R*_ in the *r*-th round, *r* = 1, …, *R*.

### Computational Complexity

#### **Theorem 3**

*Quantum algorithm U*_*R*_ *can be implemented with time complexity* ${\mathscr{O}}(\sqrt{n})$ *for the time complexity reduction of any non-reduced QG*_0_ *with an arbitrary number of L unitaries*.

**Proof**. Let

88 $${\mathscr{G}}\{|i\rangle |b\rangle |c\rangle \}$$

be a global space spanned by |*i*〉, an *n*-dimensional vector |*b*〉, and by |*c*〉, which represents the inner product state.

Particularly, the *U*_*R*_ unitary in (68) applied on an input |*φ*〉 formulated via set ${\mathscr{R}}$ of quantum registers gives

89 $$\begin{array}{rcl}|\varphi \rangle  & = & \frac{1}{\sqrt{n}}\mathop{\sum }\limits_{i=0}^{n-1}\,|{\Psi }_{i}\rangle \\  & = & \frac{1}{\sqrt{n}}\mathop{\sum }\limits_{i=0}^{n-1}\,|\partial \rangle |\eta \rangle |i\rangle |\kappa \rangle |0\rangle |0\rangle |0\rangle ,\end{array}$$

where

90 $$|{\Psi }_{i}\rangle =|\partial \rangle |\eta \rangle |i\rangle |\kappa \rangle |0\rangle |0\rangle |0\rangle ;$$

thus *U*_*R*_ can be interpreted as a rotation on an *n*-dimensional subspace ${\mathscr{S}}\{|i\rangle \}$, 0 ≤ *i* < *n*, i.e., on a span of all |*i*〉.

Let ∏ be the solution set with conditions (82) for all *i* of ∏,

91 $$\Pi =|\partial \rangle |\eta \rangle |i\rangle |\kappa \rangle |0\rangle |0\rangle |0\rangle ,$$

and let $|\varUpsilon \rangle \in {\mathscr{S}}\{|i\rangle \}$ be the superposition of all solutions:

92 $$\begin{array}{rcl}|\varUpsilon \rangle  & = & \frac{1}{\sqrt{|\Pi |}}\sum _{\xi \in \Pi }\,|\xi \rangle \\  & = & \frac{1}{\sqrt{|\Pi |}}\sum _{\xi \in \Pi }\,|\partial \rangle |\eta \rangle \sum _{\Pi }\,|i\rangle |\kappa \rangle |0\rangle |0\rangle |0\rangle .\end{array}$$

The operation *U*_*R*_ on |*φ*〉 yields the state $|{\phi }^{\ast }\rangle \in {\mathscr{S}}\{|i\rangle \}$ (see (80)):

93 $$\begin{array}{rcl}|{\phi }^{\ast }\rangle  & = & {U}_{R}(|\varphi \rangle )\\  & = & |\partial \rangle |\eta \rangle \left[(2|\psi \rangle \langle \psi |-I)\mathop{\sum }\limits_{i=0}^{n-1}\,{(-1)}^{{f}_{i}}\frac{1}{\sqrt{n}}|i\rangle \right]|\kappa \rangle |0\rangle |0\rangle |0\rangle \\  & = & \frac{1}{\sqrt{n-|\Pi |}}\sum _{|\alpha \rangle \notin \Pi }\,|\alpha \rangle \\  & = & \frac{1}{\sqrt{n-|\Pi |}}|\partial \rangle |\eta \rangle (\sum _{I-\Pi }\,|j\rangle )|\kappa \rangle |0\rangle |0\rangle |0\rangle ,\end{array}$$

thus, *U*_*R*_ is a rotation on the subspace ${\mathscr{S}}\{|{\phi }^{\ast }\rangle ,|\varUpsilon \rangle \}$ by angle ${\theta }_{{U}_{R}}$ towards (92), as

94 $${\theta }_{{U}_{R}}=2\arcsin \sqrt{\frac{|\Pi |}{n}},$$

where |∏| is the number of solutions (cardinality of solution set ∏).

*U*_*R*_ can be implemented as a rotation of ${\theta }_{{U}_{R}}$ on subspace ${\mathscr{S}}\{|i\rangle \}$ (instead of a rotation on global space (88)) via a generalized quantum searching^100^ that yields time complexity ${\mathscr{O}}(\sqrt{n})$ for an arbitrarily large quantum circuit *QG*_0_. ■

## Performance Evaluation

Assuming that the initial time complexity of the *QG*_0_ non-reduced gate structure is

95 $${\mathscr{O}}(NL),$$

where *N* is the number of *d*-dimensional (physical) quantum states in the superposed input system, and *L* is the number of unitaries in *QG*_0_, the time complexity of the reduced QG^*^ structure is

96 $${\mathscr{O}}({N}^{\ast }{L}^{\ast }),$$

where N^*^ is the number of *d*-dimensional (physical) quantum states in the reduced superposed input system, and L^*^ is the number of unitaries in the reduced gate structure QG^*^.

Since the complexity of the proposed scheme is

97 $${\mathscr{O}}(\sqrt{n}),$$

the result of (96) is a reduced time complexity with respect to (95), as the relation

98 $${N}^{\ast }{L}^{\ast } < NL-\sqrt{n},$$

holds; thus

99 $${N}^{\ast }{L}^{\ast } < NL-{d}^{N/2}.$$

The overall complexity of the QG^*^ reduced structure at the application of *U*_*R*_ is therefore

100 $${\mathscr{O}}({N}^{\ast }{L}^{\ast }+\sqrt{n})={\mathscr{O}}({N}^{\ast }{L}^{\ast }+{d}^{N\mathrm{/2}})\mathrm{}.$$

**Figure 2:**
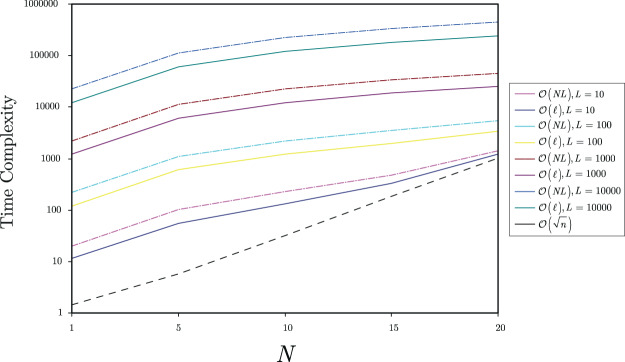
The time complexities (number of operations) for an *N*-qubit system, *d* = 2, *n* = 2^*N*^, with an initial non-reduced gate structure *QG*_0_ with *L* unitaries, *L* = {10, 100, 1000, 10000}. The time complexity of *QG*_0_ is ${\mathscr{O}}(NL)$, while ${\mathscr{O}}(\ell )$ is an upper a bound on ${\mathscr{O}}({N}^{\ast }{L}^{\ast })$ of QG^*^, ${\mathscr{O}}(\ell )={\mathscr{O}}(NL-\sqrt{n})$.

Figure 2 depicts the resulting time complexities for a qubit implementation (*N*-qubit superposed input system, and qubit gate structure with *L* unitaries).

To achieve time complexity reduction using $|\tilde{X}\rangle$ and QG^*^ instead of |*X*〉 and *QG*_0_, the relation ${\mathscr{O}}({N}^{\ast }{L}^{\ast }) < {\mathscr{O}}(\ell )={\mathscr{O}}(NL-\sqrt{n})$ must be straightforwardly satisfied, i.e., the initial complexity ${\mathscr{O}}(NL)$ has to be reduced by more than ${\mathscr{O}}(\sqrt{n})$. Since the complexity of the procedure is independent from the actual size of the gate structure, the cost remains constant ${\mathscr{O}}(\sqrt{n})$ for an arbitrarily large *L*.

## Conclusions

Gate-model quantum computers are equipped with a collection of quantum states and unitary quantum gates. The realization of the quantum circuit of a quantum computer requires high fidelity, high precision, and high-level control. Since both the timecomplexity (depth of the circuits) and the economic costs of these implementations are high in practical scenarios, a reduction of these costs is essential. Here, we defined a quantum algorithm for reducing the circuit depth of gate-model quantum computers. The method achieves a reduction in the physical layer allowing significantly reducing implementation costs. The framework is flexible and can be used for arbitrary circuit depths.

### Submission note

Parts of this work were presented in conference proceedings^101^.

### Ethics statement

This work did not involve any active collection of human data.

## Supplementary information

Supplementary Information.

## Appendix

### Algorithm 1

${\mathscr{L}}$ supervised machine learning control of ${\mathscr{C}}$

**Input**: The Ω_*i*_ coefficients from block ${\mathscr{C}}$.

**Output**: Classification of the Ω_*i*_ coefficients, error Θ of ${\mathscr{P}}$; error Δ of ${\mathscr{C}}$, updated Ω_*i*_ coefficients.

**Step 1**. Let ${{\mathscr{S}}}_{T}$ be the training set of ${\mathscr{L}}$ as ${{\mathscr{S}}}_{T}=\langle ({\kappa }_{1},{l}_{1}),\ldots ,({\kappa }_{m},{l}_{m})\rangle$, where *κ*_*i*_ is an *i*-th instance and an *n*-length vector (see (48)) drawn from the input space ${\mathscr{X}}$, while ${l}_{i}\in {{\mathscr{S}}}_{\ell }$ is the class label associated with *κ*_*i*_, where ${{\mathscr{S}}}_{\ell }$ is the set of labels ${{\mathscr{S}}}_{\ell }=\{1,\ldots ,k\}$.

**Step 2**. For *i* = 1, …, *m*, set the initial *D*_0_(*i*) distribution of the *i*-th instance of ${{\mathscr{S}}}_{T}$ as ${D}_{0}(i)=\frac{1}{m}$.

**Step 3**. Set the *R* iteration number. For an *r*-th iteration, *r* = 1, …, *R*, let *D*_*r*_ be distribution. Define hypothesis (implementable via a weak learning algorithm^102–105^) using the input distribution *D*_*r*_ as

101 $${h}_{r}:{\mathscr{X}}\to {{\mathscr{S}}}_{\ell }$$

such that the *ε*_*r*_ training error (error of *h*_*r*_) of ${\mathscr{L}}$ is

102 $${\varepsilon }_{r}={{\rm{\Pr }}}_{i}[{h}_{r}({\kappa }_{i})\ne {l}_{i}]={\sum }_{i:{h}_{r}({\kappa }_{i})\ne {l}_{i}}\,{D}_{r}(i)$$

is minimized with respect to the distribution *D*_*r*_.

**Step 4**. If *ε*_*r*_ ≤ 0.5 goto Step 5, otherwise set *R* = *r* − 1, and stop.

**Step 5**. If *h*_*r*_(*κ*_*i*_) = *l*_*i*_, set *D*_*r*+1_(*i*) as

103 $${D}_{r+1}(i)={W}_{r}\frac{{D}_{r}(i)}{{\chi }_{r}},$$

where *χ*_*r*_ is a normalization term, while *W*_*r*_ is a weighting coefficient, as

104 $${W}_{r}=\frac{{\varepsilon }_{r}}{1-{\varepsilon }_{r}}.$$

If *h*_*r*_(*κ*_*i*_) ≠ *l*_*i*_, set

105 $${D}_{r+1}(i)=\frac{{D}_{r}(i)}{{\chi }_{r}}.$$

**Step 6**. Repeat steps 3–5 for *R* times.

**Step 7**. From the *R* hypotheses *h*_1_, …, *h*_*R*_, set the final hypothesis *H* to classify the Ω_*i*_ coefficients and the input system *Y* as

106 $$H={\rm{\arg }}\mathop{{\rm{\max }}}\limits_{l\in {{\mathscr{S}}}_{\ell }}\sum _{r:{h}_{r}(\kappa )=l}\,\log \,\frac{1}{{W}_{r}},$$

and calculate the *ε*(*H*) error of *H* as

107 $$\varepsilon (H)=\sum _{i:H({\kappa }_{i})\ne {l}_{i}}\,{D}_{H}(i),$$

where *D*_*H*_ is the distribution associated to *H*.

**Step 8**. Initialize ${\partial }_{{\mathscr{C}}}$ and ${\eta }_{{\mathscr{C}}}$ parameters as

108 $${\partial }_{{\mathscr{C}}}=\frac{{\eta }_{{\mathscr{C}}}}{n},$$

where

109 $${\eta }_{{\mathscr{C}}}={\Vert Y\Vert }^{2}=Y\cdot Y=\mathop{\sum }\limits_{i=1}^{n}\,\frac{1}{n}({y}_{i}\times {y}_{i}),$$

where *Y* is defined in (28), ${y}_{i}=U({\vec{\theta }}_{i}){x}_{i}$, and

110 $${\partial }_{{\mathscr{C}}}\le \left(\frac{1}{n}({\Omega }_{i}\times {\Omega }_{i})\right)\le {\eta }_{{\mathscr{C}}},$$

where $${\Omega }_{i}=U({\vec{\theta }{\prime} }_{i}){\tilde{x}}_{i}$$.

**Step 9**. For an *i*-th coefficient Ω_*i*_, set

111 $${\partial }_{{\mathscr{C}},i}={\eta }_{{\mathscr{C}}}-\left(\frac{1}{n}({\Omega }_{i}\times {\Omega }_{i})\right).$$

**Step 10**. Set σ_*i*_ threshold for an *i*-th coefficient as

112 $${\sigma }_{i}=\frac{{\partial }_{{\mathscr{C}},i}}{{\Vert Y\Vert }^{2}}.$$

Repeat step 9, until

113 $${\sigma }_{i}\ge \varsigma $$

where *ς*, *ς* > 0 is defined via the relation

114 $$\varsigma \ge \gamma =\frac{1}{{\Vert Y\Vert }^{2}}\mathop{\sum }\limits_{i=1}^{B}\,\frac{1}{n}({\Omega }_{i}\times {\Omega }_{i}),$$

where *B* is the number of non-zero elements Ω_*i*_ of *κ*, *B* ≤ *n*. If *σ*_*i*_ < *ς*, goto step 11.

**Step 11**. Output the Θ error of ${\mathscr{P}}$ as

115 $$\Theta ={\left(\mathop{\sum }\limits_{i=1}^{B}{|\frac{1}{{\sigma }_{i}}({y}_{i}-{\Omega }_{i})|}^{\rho }\right)}^{1/\rho }+\varepsilon (H),$$

where *ρ* is a constant, while *ε*(*H*) is given in (107).

**Step 12**. Output the error Δ of ${\mathscr{C}}$ as

116 $$\Delta =\Theta -\varepsilon (H).$$

**Step 13**. According to (116), calibrate the Ω_*i*_ coefficients (defined in (49)) of output *κ* in (48) of ${\mathscr{C}}$, to yield $\Delta  < {\mathscr{E}}$ in (115), where ${\mathscr{E}}$ is a threshold on the error of ${\mathscr{P}}$.

**Procedure 1** Pre-processing ${\mathscr{P}}$

**Input**: Output *Y* (28) of the non-reduced *QG*_0_ reference circuit.

**Output**: Input $|\tilde{X}\rangle$ (31) of reduced quantum circuit *QG*^*^.

**Step 0**. Characterize the ${U}_{0}(\vec{\theta })$ (25) unitaries of the *QG*_0_ reference circuit via gate parameters (33), and the reference input |*X*〉 (22) via *X* (24). Evaluate |*Y*〉 (29) via *Y* (28).

**Step 1**. Fed *Y* into block ${\mathscr{C}}$ to determine *κ* (48), and control the results of ${\mathscr{C}}$ via ${\mathscr{L}}$.

**Step 2**. Output $\tilde{X}$ via (30) and produce $|\tilde{X}\rangle$ as (31).

### Algorithm 2

Quantum algorithm *U*_*R*_

**Input**: Output state |*Z*〉 (36) of *QG*^*^.

**Output**: Quantum state |*Y*_*R*_〉 (59).

**Step 1**. Prepare the reduced quantum state $|\tilde{X}\rangle$ (30) using $\tilde{X}$ (31) of ${\mathscr{P}}$.

**Step 2**. Prepare the unitaries $U(\vec{\theta }{\prime} )$ of (32) via $\vec{\theta }{\prime}$ (33) of *QG*^*^.

**Step 3**. Fed $|\tilde{X}\rangle$ into *QG*^*^ to yield output system |*Z*〉 (36).

**Step 4**. Utilize the quantum register set ${\mathscr{R}}$ (63). Apply *U*_*R*_ on ${\mathscr{R}}$ to get |*Y*_*R*_〉 (59) in register |*R*_6_〉 of ${\mathscr{R}}$.

## References

[1] Preskill J (2018). Quantum Computing in the NISQ era and beyond. Quantum.

[2] Arute, F. *et al*. Quantum supremacy using a programmable superconducting processor, *Nature*, Vol 574, 10.1038/s41586-019-1666-5 (2019). 10.1038/s41586-019-1666-5 31645734

[3] Harrow AW, Montanaro A (2017). Quantum Computational Supremacy. Nature.

[4] Aaronson, S. & Chen, L. Complexity-theoretic foundations of quantum supremacy experiments. *Proceedings of the 32nd Computational Complexity Conference*, CCC ’17, pages 22:1-22:67, (2017).

[5] IBM. *A new way of thinking: The IBM quantum experience*, http://www.research.ibm.com/quantum (2017).

[6] Alexeev, Y. *et al*. Quantum Computer Systems for Scientific Discovery, *arXiv:1912.07577* (2019).

[7] Loncar, M. *et al*. Development of Quantum InterConnects for Next-Generation Information Technologies, *arXiv:1912.06642* (2019).

[8] Foxen, B. *et al*. Demonstrating a Continuous Set of Two-qubit Gates for Near-term Quantum Algorithms, *arXiv:2001.08343* (2020). 10.1103/PhysRevLett.125.120504 33016760

[9] Ajagekar A, Humble T, You F (2020). Quantum Computing based Hybrid Solution Strategies for Large-scale Discrete-Continuous Optimization Problems. Computers and Chemical Engineering.

[10] Ajagekar A, You F (2019). Quantum computing for energy systems optimization: Challenges and opportunities. Energy.

[11] Harrigan, M. *et al*. Quantum Approximate Optimization of Non-Planar Graph Problems on a Planar Superconducting Processor, *arXiv:2004.04197v1* (2020).

[12] Rubin, N. *et al*. Hartree-Fock on a superconducting qubit quantum computer, *arXiv:2004.04174v1* (2020). 10.1126/science.abb9811 32855334

[13] Lloyd, S. Quantum approximate optimization is computationally universal, *arXiv:1812.11075* (2018).

[14] Shor, P. W. Algorithms for quantum computation: discrete logarithms and factoring. In: *Proceedings of the 35th Annual Symposium on Foundations of Computer Science* (1994).

[15] Debnath S (2016). Demonstration of a small programmable quantum computer with atomic qubits. Nature.

[16] Barends R (2014). Superconducting quantum circuits at the surface code threshold for fault tolerance. Nature.

[17] Ofek N (2016). Extending the lifetime of a quantum bit with error correction in superconducting circuits. Nature.

[18] Kielpinski D, Monroe C, Wineland DJ (2002). Architecture for a large-scale ion-trap quantum computer. Nature.

[19] Gyongyosi, L. Quantum State Optimization and Computational Pathway Evaluation for Gate-Model Quantum Computers, *Scientific Reports*, 10.1038/s41598-020-61316-4 (2020). 10.1038/s41598-020-61316-4 PMC7066182 32161308

[20] Gyongyosi, L. & Imre, S. Training Optimization for Gate-Model Quantum Neural Networks, *Scientific Reports*, 10.1038/s41598-019-48892-w (2019). 10.1038/s41598-019-48892-w PMC6722103 31481737

[21] Gyongyosi, L. & Imre, S. Dense Quantum Measurement Theory, *Scientific Reports*, 10.1038/s41598-019-43250-2 (2019). 10.1038/s41598-019-43250-2 PMC6494868 31043650

[22] Gyongyosi, L. & Imre, S. State Stabilization for Gate-Model Quantum Computers, *Quantum Information Processing*, 10.1007/s11128-019-2397-0, (2019).

[23] Gyongyosi, L. & Imre, S. Quantum Circuit Design for Objective Function Maximization in Gate-Model Quantum Computers, *Quantum Information Processing*, 10.1007/s11128-019-2326-2 (2019).

[24] Brandao, F. G. S. L., Broughton, M., Farhi, E., Gutmann, S. & Neven, H. For Fixed Control Parameters the Quantum Approximate Optimization Algorithm’s Objective Function Value Concentrates for Typical Instances, *arXiv:1812.04170* (2018).

[25] Zhou, L.,Wang, S.-T., Choi, S., Pichler, H. & Lukin, M. D. Quantum Approximate Optimization Algorithm: Performance, Mechanism, and Implementation on Near-Term Devices, arXiv:1812.01041 (2018).

[26] Lechner, W. Quantum Approximate Optimization with Parallelizable Gates, *arXiv:1802.01157v2* (2018).

[27] Crooks, G. E. Performance of the Quantum Approximate Optimization Algorithm on the Maximum Cut Problem, *arXiv:1811.08419* (2018).

[28] Ho, W. W., Jonay, C. & Hsieh, T. H. Ultrafast State Preparation via the Quantum Approximate Optimization Algorithm with Long Range Interactions, *arXiv:1810.04817* (2018).

[29] Song C (2017). 10-Qubit Entanglement and Parallel Logic Operations with a Superconducting Circuit. Physical Review Letters.

[30] Laurenza R, Pirandola S (2017). General bounds for sender-receiver capacities in multipoint quantum communications. Phys. Rev. A.

[31] Gyongyosi L, Imre S, Nguyen HV (2018). A Survey on Quantum Channel Capacities. IEEE Communications Surveys and Tutorials.

[32] Imre, S. & Gyongyosi, L. *Advanced Quantum Communications - An Engineering Approach*. Wiley-IEEE Press (New Jersey, USA), (2012).

[33] Shor PW (1995). Scheme for reducing decoherence in quantum computer memory. Phys. Rev. A.

[34] Petz, D. *Quantum Information Theory and Quantum Statistics*, Springer-Verlag, Heidelberg, (2008).

[35] Bacsardi L (2013). On the Way to Quantum-Based Satellite Communication. IEEE Comm. Mag..

[36] Gyongyosi, L. & Imre, S. Entanglement-Gradient Routing for Quantum Networks, *Sci. Rep*., Nature, 10.1038/s41598-017-14394-w (2017). 10.1038/s41598-017-14394-w PMC5660278 29079759

[37] Gyongyosi, L. & Imre, S. Entanglement Availability Differentiation Service for the Quantum Internet, *Sci. Rep*., Nature, 10.1038/s41598-018-28801-3 (2018). 10.1038/s41598-018-28801-3 PMC6045587 30006577

[38] Gyongyosi, L. & Imre, S. Multilayer Optimization for the Quantum Internet, *Sci. Rep*., Nature, (2018). 10.1038/s41598-018-30957-x PMC6107661 30140031

[39] Gyongyosi, L. & Imre, S. Decentralized Base-Graph Routing for the Quantum Internet, *Phys. Rev. A*, American Physical Society, (2018).

[40] Gyongyosi, L. & Imre, S. A Survey on Quantum Computing Technology, *Computer Science Review*, Elsevier, 10.1016/j.cosrev.2018.11.002, ISSN: 1574-0137, (2018).

[41] Farhi, E., Goldstone, J. & Gutmann, S. A Quantum Approximate Optimization Algorithm. *arXiv:1411.4028*. (2014).

[42] Farhi, E., Goldstone, J., Gutmann, S. & Neven, H. Quantum Algorithms for Fixed Qubit Architectures. *arXiv:1703.06199v1* (2017).

[43] Farhi, E. & Neven, H. Classification with Quantum Neural Networks on Near Term Processors, *arXiv:1802.06002v1* (2018).

[44] Farhi, E., Goldstone, J., Gutmann, S. & Zhou, L. The Quantum Approximate Optimization Algorithm and the Sherrington-Kirkpatrick Model at Infinite Size, *arXiv:1910.08187* (2019).

[45] Farhi, E., Goldstone, J. & Gutmann, S. A Quantum Approximate Optimization Algorithm Applied to a Bounded Occurrence Constraint Problem. *arXiv:1412.6062*. (2014).

[46] Pirandola S (2019). End-to-end capacities of a quantum communication network. Commun. Phys..

[47] Pirandola S, Laurenza R, Ottaviani C, Banchi L (2017). Fundamental limits of repeaterless quantum communications. Nature Communications.

[48] Pirandola S (2018). Theory of channel simulation and bounds for private communication. Quantum Sci. Technol..

[49] Van Meter, R. *Quantum Networking*, John Wiley and Sons Ltd, ISBN 1118648927, 9781118648926 (2014).

[50] Biamonte J (2017). Quantum Machine Learning. Nature.

[51] LeCun Y, Bengio Y, Hinton G (2014). Deep Learning. Nature.

[52] Monz T (2016). Realization of a scalable Shor algorithm. Science.

[53] Goodfellow, I., Bengio, Y. & Courville, A. *Deep Learning*. MIT Press. Cambridge, MA, (2016).

[54] Rebentrost, P., Mohseni, M. & Lloyd, S. Quantum Support Vector Machine for Big Data Classification. *Phys. Rev. Lett*. **113** (2014). 10.1103/PhysRevLett.113.130503 25302877

[55] Lloyd, S. The Universe as Quantum Computer, *A Computable Universe: Understanding and exploring Nature as computation*, Zenil, H. ed., World Scientific, Singapore, 2012, *arXiv:1312.4455v1* (2013).

[56] Lloyd, S., Mohseni, M. & Rebentrost, P. Quantum algorithms for supervised and unsupervised machine learning, *arXiv:1307.0411v2* (2013).

[57] Lloyd, S., Garnerone, S. & Zanardi, P. Quantum algorithms for topological and geometric analysis of data. *Nat. Commun*., **7**, *arXiv:1408.3106* (2016). 10.1038/ncomms10138 PMC4737711 26806491

[58] Lloyd S (2004). Infrastructure for the quantum Internet. ACM SIGCOMM Computer Communication Review.

[59] Lloyd S, Mohseni M, Rebentrost P (2014). Quantum principal component analysis. Nature Physics.

[60] Schuld, M., Sinayskiy, I. & Petruccione, F. An introduction to quantum machine learning. *Contemporary Physics* 56, pp. 172–185. *arXiv: 1409.3097* (2015).

[61] Krisnanda T (2018). Probing quantum features of photosynthetic organisms. npj Quantum Inf..

[62] Krisnanda T (2017). Revealing Nonclassicality of Inaccessible Objects. Phys. Rev. Lett..

[63] Krisnanda T (2020). Observable quantum entanglement due to gravity. npj Quantum Inf..

[64] Krisnanda T (2018). Detecting nondecomposability of time evolution via extreme gain of correlations. Phys. Rev. A.

[65] Shannon, K., Towe, E. & Tonguz, O. On the Use of Quantum Entanglement in Secure Communications: A Survey, *arXiv:2003.07907*, (2020).

[66] Amoretti, M. & Carretta, S. Entanglement Verification in Quantum Networks with Tampered Nodes, *IEEE Journal on Selected Areas in Communications*, 10.1109/JSAC.2020.2967955 (2020).

[67] Cao, Y. *et al*. Multi-Tenant Provisioning for Quantum Key Distribution Networks with Heuristics and Reinforcement Learning: A Comparative Study, *IEEE Transactions on Network and Service Management*, 10.1109/TNSM.2020.2964003 (2020).

[68] Cao, Y. *et al*. Key as a Service (KaaS) over Quantum Key Distribution (QKD)-Integrated Optical Networks, *IEEE Comm. Mag*., 10.1109/MCOM.2019.1701375 (2019).

[69] Farhi, E., Gamarnik, D. & Gutmann, S. The Quantum Approximate Optimization Algorithm Needs to See the Whole Graph: A Typical Case, *arXiv:2004.09002v1* (2020).

[70] Farhi, E., Gamarnik, D. & Gutmann, S. The Quantum Approximate Optimization Algorithm Needs to See the Whole Graph: Worst Case Examples, *arXiv:2005.08747* (2020).

[71] Brown, K. A. & Roser, T. Towards storage rings as quantum computers. *Phys. Rev. Accel. Beams* **23**, 054701 (2020).

[72] Sax, I. *et al*. Approximate Approximation on a Quantum Annealer, *arXiv:2004.09267* (2020).

[73] Pirandola S, Braunstein SL (2016). Unite to build a quantum internet. Nature.

[74] Wehner S, Elkouss D, Hanson R (2018). Quantum internet: A vision for the road ahead. Science.

[75] Pirandola S (2019). Bounds for multi-end communication over quantum networks. Quantum Sci. Technol..

[76] Pirandola, S. Capacities of repeater-assisted quantum communications, *arXiv:1601.00966* (2016).

[77] Pirandola, S. *et al*. Advances in Quantum Cryptography, *arXiv:1906.01645* (2019).

[78] Cacciapuoti, A. S. *et al*. Quantum Internet: Networking Challenges in Distributed Quantum Computing, *arXiv:1810.08421* (2018).

[79] Caleffi, M. End-to-End Entanglement Rate: Toward a Quantum Route Metric, *2017 IEEE Globecom*, 10.1109/GLOCOMW.2017.8269080, (2018).

[80] Caleffi, M. Optimal Routing for Quantum Networks, *IEEE Access*, Vol 5, 10.1109/ACCESS.2017.2763325 (2017).

[81] Caleffi, M., Cacciapuoti, A. S. & Bianchi, G. Quantum Internet: from Communication to Distributed Computing, *aXiv:1805.04360* (2018).

[82] Castelvecchi, D. The quantum internet has arrived, *Nature*, News and Comment, https://www.nature.com/articles/d41586-018-01835-3 (2018). 10.1038/d41586-018-01835-3 29446394

[83] Cuomo, D., Caleffi, M. & Cacciapuoti, A. S. Towards a Distributed Quantum Computing Ecosystem, *arXiv:2002.11808v1* (2020).

[84] Chakraborty, K., Rozpedeky, F., Dahlbergz, A. & Wehner, S. Distributed Routing in a Quantum Internet, *arXiv:1907.11630v1* (2019).

[85] Khatri S, Matyas CT, Siddiqui AU, Dowling JP (2019). Practical figures of merit and thresholds for entanglement distribution in quantum networks. Phys. Rev. Research.

[86] Kozlowski, W. & Wehner, S. Towards Large-Scale Quantum Networks, *Proc. of the Sixth Annual ACM International Conference on Nanoscale Computing and Communication*, Dublin, Ireland, *arXiv*:1909.08396 (2019).

[87] Pathumsoot, P., Matsuo, T., Satoh, T., Hajdusek, M., Suwanna, S. & Van Meter, R. Modeling of Measurement-based Quantum Network Coding on IBMQ Devices. *Phys. Rev. A* **101**, 052301 (2020).

[88] Pal, S., Batra, P., Paterek, T. & Mahesh, T. S. Experimental localisation of quantum entanglement through monitored classical mediator, *arXiv:1909.11030v1* (2019).

[89] Miguel-Ramiro, J. & Dur, W. Delocalized information in quantum networks, *New J. Phys*, 10.1088/1367-2630/ab784d (2020).

[90] Pirker, A. & Dur, W. A quantum network stack and protocols for reliable entanglement-based networks, *arXiv:1810.03556v1* (2018).

[91] Miguel-Ramiro, J., Pirker, A. & Dur, W. Genuine quantum networks: superposed tasks and addressing, *arXiv:2005.00020v1* (2020).

[92] Amer, O., Krawec, W. O. and Wang, B. Efficient Routing for Quantum Key Distribution Networks, *arXiv:2005.12404* (2020).

[93] Liu, Y. Preliminary Study of Connectivity for Quantum Key Distribution Network, *arXiv:2004.11374v1* (2020).

[94] Hosen MA, Khosravi A, Nahavandi S, Creighton D (2015). IEEE Transactions on Industrial Electronics.

[95] Precup R-E, Angelov P, Costa BSJ, Sayed-Mouchaweh M (2015). An overview on fault diagnosis and nature-inspired optimal control of industrial process applications. Computers in Industry.

[96] Saadat J, Moallem P, Koofigar H (2017). Training echo state neural network using harmony search algorithm. International Journal of Artificial Intelligence.

[97] Vrkalovic S, Lunca E-C, Borlea I-D (2018). Model-free sliding mode and fuzzy controllers for reverse osmosis desalination plants. International Journal of Artificial Intelligence.

[98] Guerreschi, G. G. & Smelyanskiy. M. Practical optimization for hybrid quantum-classical algorithms, *arXiv:1701.01450* (2017).

[99] Pang, C.-Y., Zhou, Z.-W. & Guo, G.-C. Quantum Discrete Cosine Transform for Image Compression, *arXiv:quant-ph/0601043* (2006).

[100] Grover, L. K. A fast quantum mechanical algorithm for database search, *Proceedings of the 28th ACM symposium on Theory of Computing (STOC)*, pp.212–218 (1996).

[101] Gyongyosi, L. A Universal Quantum Algorithm for Time Complexity Reduction of Quantum Computers, *Proceedings of Quantum Information Processing 2019* (QIP 2019), University of Colorado Boulder, USA (2019).

[102] Roy, A. & Chakraborty, R. S. Camera Source Identification Using Discrete Cosine Transform Residue Features and Ensemble Classifier, *2017 IEEE Conference on Computer Vision and Pattern Recognition Workshops (CVPRW)*, 10.1109/CVPRW.2017.231 (2017).

[103] Freund, Y. & Schapire, R. E. Experiments with a new boosting algorithm, *ICML*, vol. 96, pp. 148–156. 4 (1996).

[104] Freund Y, Schapire RE (1997). A decision-theoretic generalization of on-line learning and an application to boosting. Journal of Computer and System Sciences..

[105] Criminisi, A., Shotton, J. & Konukoglu, E. Decision forests for classification, regression, density estimation, manifold learning and semi-supervised learning, *Microsoft Research Cambridge, Tech. Rep*. MSRTR-2011-114, vol. 5, no. 6, p. 12, 3 (2011).

