# Supplementary material for: Circuit Depth Reduction for Gate-Model Quantum Computers
Source: Sci Rep. 2020 Jul 8;10:11229. doi: 10.1038/s41598-020-67014-5 (PMC7343887; doi:10.1038/s41598-020-67014-5)
Supplement: Supplementary file 1 — Supplementary Information. [file 41598_2020_67014_MOESM1_ESM.pdf]

Circuit Depth Reduction for Gate-Model Quantum Computers

Laszlo Gyongyosi[1,2,3,*] **and Sandor Imre**[2]

[1]School of Electronics and Computer Science, University of Southampton, Southampton, SO17 1BJ, UK
[2]Department of Networked Systems and Services, Budapest University of Technology and Economics, Budapest, H-1117 Hungary
[3]MTA-BME Information Systems Research Group, Hungarian Academy of Sciences, Budapest, H-1051 Hungary
[*]gyongyosi@hit.bme.hu

ABSTRACT

Quantum computers utilize the fundamentals of quantum mechanics to solve computational problems more efficiently than traditional computers. Gate-model quantum computers are fundamental to implement near-term quantum computer architectures and quantum devices. Here, a quantum algorithm is defined for the circuit depth reduction of gate-model quantum computers. The proposed solution evaluates the reduced time complexity equivalent of a reference quantum circuit. We prove the complexity of the quantum algorithm and the achievable reduction in circuit depth. The method provides a tractable solution to reduce the time complexity and physical layer costs of quantum computers.

A Appendix

A.1 Notations

The notations of the manuscript are summarized in Table A.1.

Table A.1. Summary of notations.

Notation	*Description*
QG_0	Reference (non-reduced time complexity) quantum gate structure of a quantum computer.
QG^*	Reduced time complexity quantum gate structure of a quantum computer.
$\|X\rangle$	Superposed input system of the non-reduced QG_0 gate structure, $\|X\rangle = \frac{1}{\sqrt{d^N}}\sum_{x_i=1}^{d^N}\|x_i\rangle$, where N is the number of the d-dimensional quantum states that formulate $\|X\rangle$, and $\|x_i\rangle$ is the i-th input vector, $i = 1,\ldots,n$, $n = d^N$.
d	Dimension of the quantum state.
N	Number of d-dimensional quantum states in the input system.
n	Number of vectors in the input system of N quantum states, $n = d^N$.
L	Number of unitary gates in the QG structure of the quantum computer.
$U_i(\theta_i)$	An i-th unitary gate, $U_i(\theta_i) = \exp(-i\theta_i P_i)$, where P_i is a generalized Pauli operator formulated by a tensor product of Pauli operators $\{\sigma_x, \sigma_y, \sigma_z\}$, while θ_i is referred to as the gate parameter associated to $U_i(\theta_i)$.
$U(\vec{\theta})$	System state of the quantum computer, $U(\vec{\theta}) = U_L(\theta_L)U_{L-1}(\theta_{L-1})\ldots U_1(\theta_1)$, where $U_i(\theta_i)$ identifies an i-th unitary gate.
$\vec{\theta}$	Gate parameter vector, a collection of gate parameters of the L unitaries, $\vec{\theta} = (\theta_1,\ldots,\theta_{L-1},\theta_L)^T$.

C	Classical objective function of a computational problem fed into the quantum computer.
$\mathscr{C}$	Computational block in the $\mathscr{P}$ pre-processing phase, outputs κ.
P	Generalized Pauli operator formulated by the tensor product of Pauli operators $\{\sigma_x, \sigma_y, \sigma_z\}$.
$\mathscr{P}$	Pre-processing in the logical layer, $\mathscr{P} = \mathscr{C}\mathscr{L}$, where $\mathscr{C}$ is a computational block, while $\mathscr{L}$ is a machine learning control block to calibrate the results of $\mathscr{C}$.
$\mathscr{L}$	Machine learning control unit in the $\mathscr{P}$ pre-processing, outputs the Δ error for feedback control.
X	Classical representation of $\|X\rangle$, $X = \frac{1}{\sqrt{d^N}}\left(x_1,\ldots,x_n\right)^T$, where x_i is the classical representation of $\|x_i\rangle$.
$U_0(\vec{\theta})$	Non-reduced gate structure matrix of QG_0, $U_0(\vec{\theta}) = (U_0(\vec{\theta}_1)\ldots U_0(\vec{\theta}_n))$.
$U_0(\vec{\theta}_i)$	Unitary sequence associated to $\|x_i\rangle$ in QG_0, $U_0(\vec{\theta}_i) = U_L\left(\theta_{i,L}\right)U_{L-1}\left(\theta_{i,L-1}\right)\ldots U_1\left(\theta_{i,1}\right)$.
Y	An n-dimensional output vector, $Y = \frac{1}{\sqrt{d^N}}\left(y_1,\ldots,y_n\right)^T$.
$\|Y\rangle$	Output quantum state of the non-reduced QG_0 structure, $\|Y\rangle = U_0(\vec{\theta})\|X\rangle$.
$U(\vec{\theta}')$	Reduced gate structure matrix of QG^*, $U(\vec{\theta}') = (U(\vec{\theta}'_{n^*})U(\vec{\theta}'_{n^*-1})\ldots U(\vec{\theta}'_1))$, where $U(\vec{\theta}'_i)$ is the reduced unitary sequence associated to $\|\tilde{x}_i\rangle$.
$U(\vec{\theta}'_i)$	Reduced unitary sequence associated with a reduced input $\|\tilde{x}_i\rangle$ in QG^*, $U(\vec{\theta}'_i) = U_L(\tilde{\theta}_{i,L})U_{L-1}(\tilde{\theta}_{i,L-1})\ldots U_1(\tilde{\theta}_{i,1})$.
$\tilde{X}$	Classical representation of the reduced quantum state $\|\tilde{X}\rangle$ fed into QG^*, $\tilde{X} = \frac{1}{\sqrt{d^{N^*}}}\left(\tilde{x}_1,\ldots,\tilde{x}_{n^*}\right)^T$.
$\|\tilde{X}\rangle$	Reduced quantum state $\|\tilde{X}\rangle$ fed into QG^*, $\|\tilde{X}\rangle = \frac{1}{\sqrt{n^*}}\sum_{\tilde{x}_i=1}^{n^*}\|\tilde{x}_i\rangle$, where $n^* = d^{N^*}$.
N^*	Number of d-dimensional (physical) quantum states that formulate $\|\tilde{X}\rangle$.
Z	Classical representation of the $\|Z\rangle$ output QG^*, $Z = U(\vec{\theta}')\tilde{X}$.
$\|Z\rangle$	Output quantum state $\|Z\rangle$ of QG^*, $\|Z\rangle = U(\vec{\theta}')\|\tilde{X}\rangle = \frac{1}{\sqrt{n^*}}\sum_{\tilde{x}_i=1}^{n^*}U(\vec{\theta}'_i)\|\tilde{x}_i\rangle$.
U_R	Quantum algorithm for the recovery of reference output quantum state $\|Y\rangle$ of QG_0 from the output $\|Z\rangle$ of QG^*.
$\|Y_R\rangle$	Recovered output quantum state via U_R.
G	Matrix of n coefficients, a_i, $i = 1,\ldots,n$, $G = \begin{pmatrix}a_1 & \cdots & a_n\end{pmatrix}^T$.
$\Sigma\theta_i$	Sum of L gate parameters, $\Sigma\theta_i = \sum_{j=1}^{L}\theta_{i,j}$, where $\theta_{i,j}$ identifies the gate parameter of a j-th unitary $U_{i,j}(\theta)$ associated to an i-th input x_i, while unitary sequence $U_0(\vec{\theta}_i)$ to an i-th input x_i, is as $U_0(\vec{\theta}_i) = \exp(i\Sigma\theta_i B_i)$, where B_i is a generalized Pauli operator.
c_p	A coefficient of $\mathscr{C}$.
f_i	A coefficient of $\mathscr{C}$.
A_p	A coefficient of $\mathscr{C}$.
γ	Matrix of n coefficients, defined via $\mathscr{C}$, $\gamma = \left(c_1, c_2, \ldots, c_n\right)^T$.
$\cdot$	Inner product.

ς_i	An n-length vector of $\mathscr{C}$.
κ	An n-length vector $\kappa = \frac{1}{\sqrt{n}} (\Omega_1, \ldots, \Omega_n)^T$.
Ω_i	An i-th component of κ, defined as $\Omega_i = U(\vec{\theta}_i')\tilde{x}_i = U_L(\tilde{\theta}_{i,L})U_{L-1}(\tilde{\theta}_{i,L-1}) \ldots U_1(\tilde{\theta}_{i,1})\tilde{x}_i$.
$\lvert\tilde{x}_p\rangle$	A p-th input $\lvert\tilde{x}_p\rangle$ of the reduced structure QG^*.
$U(\vec{\theta}_p')$	Reduced quantum gate sequence of QG^*.
$\Sigma\tilde{\theta}_p$	Sum of gate parameters in QG^*.
H	Hypothesis from $\mathscr{L}$.
Θ	Error of $\mathscr{P}$.
Δ	Error of $\mathscr{C}$ in $\mathscr{P}$.
$\mathscr{S}_T$	Training set of $\mathscr{L}$, $\mathscr{S}_T = \langle(\kappa_1, l_1), \ldots, (\kappa_m, l_m)\rangle$, $\mathscr{S}_T = \langle(\kappa_1, l_1), \ldots, (\kappa_m, l_m)\rangle$, where κ_i is an i-th instance and an n-length vector, while $l_i \in \mathscr{S}_\ell$ is the class label associated with κ_i, where $\mathscr{S}_\ell$ is the set of labels $\mathscr{S}_\ell = \{1, \ldots, k\}$.
$\mathscr{S}_\ell$	Set of labels for $\mathscr{L}$.
D	A distribution parameter for $\mathscr{L}$.
R	Iteration number for $\mathscr{L}$.
$\mathscr{X}$	Input space of κ.
ε_r	Training error (error of h_r) of $\mathscr{L}$.
χ_r	Normalization term.
$\varepsilon(H)$	Error of H.
∂_C, η_C	Parameters for $\mathscr{P}$.
σ_i	Threshold parameter for $\mathscr{P}$.
$\mathscr{E}$	Threshold on the error of $\mathscr{P}$.
$\lvert\Phi_i\rangle$	Quantum state, subject to be determined via U_R as an inner product state $\lvert\Phi_i\rangle = \lvert\omega_i \cdot \kappa\rangle$.
ω_i	An n-length vector, $\omega_i = (\omega_{i,1}, \ldots, \omega_{i,n})^T$, defined for a given j, as $\omega_j = \left(e^{i\left(\Sigma\theta_1 - \Sigma\tilde{\theta}_1\right)} A_j \cos\frac{j\pi}{2n}, \ldots, e^{i\left(\Sigma\theta_n - \Sigma\tilde{\theta}_n\right)} A_j \cos\frac{(2n-1)j\pi}{2n}\right)^T$, where $\Sigma\theta_i$ is as $\Sigma\theta_i = \sum_{i=0}^{n-1} A_p \left(\Sigma\tilde{\theta}_p\right) \cos\frac{\pi(2i+1)p}{2n}$.
$W\kappa$	Matrix, $W\kappa = (\omega_1 \cdot \kappa, \ldots, \omega_n \cdot \kappa)^T$.
$\lvert Y_R\rangle$	Recovered quantum state outputted by U_R, $\lvert Y_R\rangle = \sum_{i=1}^n \lvert\Phi_i\rangle = \sum_{i=1}^n \lvert\omega_i \cdot \kappa\rangle$.
$\mathscr{R}$	Set $\mathscr{R}$ of quantum registers, $U_R : \mathscr{R} = \{\lvert R_1\rangle, \ldots, \lvert R_7\rangle\}$, where $\lvert R_i\rangle$ is an i-th quantum register.
∂, η	Parameters of U_R.
U_S	A unitary of U_R.
$\lvert\Psi_S\rangle$	Eigenstate of U_S, $\lvert\Psi_S\rangle = \lvert\partial\rangle\lvert\eta\rangle\lvert i\rangle\lvert\kappa\rangle\lvert 0\rangle\lvert 0\rangle\lvert 0\rangle$.
U_0	A unitary operator, prepares $\lvert R_5\rangle = \lvert\omega_i\rangle$ for a given index state $\lvert R_4\rangle = \lvert i\rangle$ from an initial $\lvert R_4\rangle\lvert R_5\rangle = \lvert i\rangle\lvert 0\rangle$, as $U_0(\lvert i\rangle\lvert 0\rangle) = \lvert 0 \oplus \omega_i\rangle$, where $\oplus$ is the CNOT operation.
O_{Φ_i}	Oracle to compute Φ_i.

O_{f_i}	Oracle, outputs function f_i.
f_i	Function, outputted by O_{f_i}, defined as $f_i = \begin{cases} 1, \text{ if } \partial \leq \left(\frac{1}{\sqrt{n}} y_i\right)^2 \leq \eta \\ 0, \text{ otherwise} \end{cases}$.
$(-1)^{f_i}$	Eigenvalue of U_S.
$\|\phi_0\rangle$	Input state for U_R.
k	Iteration number for the application of U_R.
O_Z	An oracle to prepare register state $\|R_6\rangle = \|\Phi_i\rangle = \|\omega_i \cdot \kappa\rangle$.
$\|\phi^*\rangle$	Output system state, $\|\phi^*\rangle = O_Z U_0 (\|\phi_k\rangle)$, where $\|\phi_k\rangle = (U_R)^k \|\phi_0\rangle$.
$\partial^{(r)}$, $\eta^{(r)}$	Parameters for the r-th repetition round of U_R, $r = 1, \ldots, R$, where R is the total number of repetitions.
$\Phi_i^{(r)}$	Measured value of $\|\Phi_i\rangle$ in the r-th repetition of U_R.
z_R	Classical string, results from the M measurement of $\|Y_R\rangle$.
z	Classical string, results from the M measurement of $\|Y\rangle$.
$C(z_R)$	Objective function value evaluated via z_R.
$C(z)$	Objective function value evaluated via z.
$\mathscr{D}$	Distortion coefficient, defined as $\mathscr{D} = \|C(z) - C(z_R)\|$.
$\mathscr{D}^{(R)}$	Averaged distortion coefficient via R measurement rounds, $\mathscr{D}^{(R)} = \frac{1}{R}\sum_{r=1}^{R} \left\| C^{(r)}(z) - C^{(r)}(z_R) \right\|$, where $C^{(r)}(z)$ and $C^{(r)}(z_R)$ are the objective function values associated to z and z_R in the r-th round, $r = 1, \ldots, R$.
$\mathscr{G}\{\|i\rangle\|b\rangle\|c\rangle\}$	A global space spanned by $\|i\rangle$, an n-dimensional vector $\|b\rangle$, and by $\|c\rangle$ that represents the inner product state.
$\|\varphi\rangle$	Input quantum state, formulated via the set $\mathscr{R}$ of quantum registers.
$\mathscr{S}\{\|i\rangle\}$	An n-dimensional subspace, $0 \leq i < n$.
Π	Solution set.
$\|\Upsilon\rangle$	Superposition of all solutions.
θ_{U_R}	Angle of rotation on the subspace $\mathscr{S}\{\|\phi^*\rangle, \|\Upsilon\rangle\}$ by U_R, $\theta_{U_R} = 2 \arcsin \sqrt{\frac{\|\Pi\|}{n}}$, where $\|\Pi\|$ is the number of solutions (cardinality of solution set Π).
$\mathscr{O}(NL)$	Initial time complexity of the QG_0 non-reduced gate structure, where N is the number of d-dimensional (physical) quantum states in the superposed input system, and L is the number of unitaries in QG_0.
$\mathscr{O}(N^* L^*)$	Time complexity of the reduced QG^* structure, where N^* is the number of d-dimensional (physical) quantum states in the reduced superposed input system, L^* is the number of unitaries in the reduced gate structure QG^*.
$\mathscr{O}(\sqrt{n})$	Complexity of the proposed framework of U_R (the $\mathscr{P}$ pre-processing is not implemented in the physical layer).
$\mathscr{O}(\ell)$	An upper a bound on $\mathscr{O}(N^* L^*)$, as $\mathscr{O}(\ell) = \mathscr{O}(NL - \sqrt{n})$ with a target relation $\mathscr{O}(N^* L^*) < \mathscr{O}(\ell)$.
